# The Pathology and Treatment of Venereal Diseases Including the Results of Recent Investigations on the Subject

**Published:** 1861-11

**Authors:** 


THE PATHOLOGY AND TREATMENT OF VENEREAL DISEASES; including the Results of Recent Investigations on the Subject. By FREEMAN J. BUMSTEAD, M. D., Lecturer on Venereal Diseases, at the College of Physicians and Surgeons, New York; Surgeon to St. Luke's Hospital; Assistant-Surgeon to the New York Eye Infirmary. 8vo., pp. 685. Philadelphia: Blanchard & Lea. 1861. Chicago: W. B. Keen.

The views of the profession in regard to the pathology of syphilis, have undergone important changes during the last five years. Fanciful theories, which distorted the observations of earlier writers, have given way to the sober impressions of facts. Men doubt, where they once blindly credited, and inquire into what they formerly took for granted. One is particularly struck with the recent radical changes which have taken place in the views of the profession, concerning the varieties of chancre, inoculation, transmission of secondary symptoms, period of incubation, etc., after perusing our author.

Dr. BUMSTEAD has shown himself more than a compiler in this volume; for whilst he has given us a complete summary of the labor of others in this specialty, he has proved himself to possess a sound judgment in the selection of material, and a very extensive knowledge of his subject. He is favorably known to the profession by a series of lectures upon gonorrhœa and syphilis, delivered before the class of the College of Physicians and Surgeons, and published in the *N. Y. Jour. of Med.*, 1859.

The volume is divided into the subject of gonorrhœa and its complications—including the anatomy, pathology and treatment of stricture—and the chancroid—its complications and syphilis. The introduction is devoted, chiefly, to a discussion of the origin of venereal diseases, and the distinct natures of gonorrhœa and syphilis. It comprises a condensed and interesting epitome of all the medical literature pertaining to the origin of venereal diseases, and need not detain us, except to remark that the author does not regard the testimony in favor of the supposed American origin of syphilis as at all conclusive; and that gonorrhœa and syphilis are due to different poisons; although inoculations,—relied upon as conclusive evidence of this fact, by Ricord—have been proved fallacious, yet other facts and observations sufficiently sustain the theory.* No allusion is made to the well-known experiments of Hunter.

Amongst the causes of gonorrhœa, the author admits its origin in coitus with women suffering from leucorrhœa as an accident of frequent occurrence; and as often due to accidental causes, and not to direct contagion. As proof of the contagious character of a leucorrhœal discharge, he cites as an example the ophthalmia of new born-children, its origin, and virulence. Ricord, Diday, Fournier, Skey and others, are quoted as authorities, all of whom maintain that *gonor-*

---

*To Mr. Ricord is due the credit of having introduced a new method of investigating the nature of the venereal diseases; namely, that of inoculating the products of the different affections which arise, with the point of a lancet on some apparently healthy part of the skin of the affected person. By this method of investigation, M. Ricord arrives at the following, among other conclusions: * * * * * "*That blenorrhagia* (*or gonorrhœa*) *and chancre, are two affections, entirely distinct.*"

The concurrent testimony of all observers has now ceded to Ricord his first point, namely, that the pus derived from a chancre, contains a very powerful agent which escapes detection by our senses, and which is not found in the pus of blenorrhagin; when this subtle and occult agent is inoculated, the morbid process which ensues, may be traced in the most satisfactory manner. If the pus from a suppurating chancre, duiing its period of progress, or during the time that it remains stationary, be inoculated, the following succession of events may (according to M. Ricord,) be observed. * * * * "From the fifth day, the subjacent tissue, which had before undergone no change, beyond being slightly œdematous, becomes infiltrated by a plastic lymph, which affords to the touch the peculiar hard and elastic feeling of certain forms of cartilage. * * * (It does not appear how M. Ricord became acquainted with this fact. According to his own published opinion, the process of induration could only take place in those who had not previously had constitutional syphilis; and these, M. Ricord has always, with great propriety, refused to inoculate.)—*Henry Lee.*

Our author truly remarks, also, that "the true chancre is precisely upon the same footing as gonorrhœa; neither the one or the other is auto-inoculable; and hence this test at one time, much insisted upon by Ricord, though not original with him, is proved fallacious." Vidal is one of the latest writers on the opposite side, and maintains that certain persons are naturally incapable of being inoculated with the syphilitic virus; and that in others this immunity may be acquired under certain circumstances. Our author states that "Diday has adduced the testimony of three of the Internes of the Hôpital du Midi in proof of the fact that Vidal invariably treated blenorrhgia as a simple inflammation, without mercury."

*rhœa may arise from intercourse with women, who, themselves, have not the disease.* The importance of this truth, in a medico-legal point of view, is apparent. The writer has had under treatment, within a fortnight, as severe a case of gonorrhœa as is often seen, accompanied with severe chordee, in a patient whose word could be implicitly relied upon, and who had intercourse with no other female than his wife. She was examined, and no evidence of a venereal disease was present, but she had had the "whites" at the time the husband contracted his gonorrhœa.

Space will not permit any quotations from the chapter upon *stricture* and its treatment, which seems to be all that can be desired in a work of this kind; and we pass on to the one on *syphilis.* He divides the primary ulcer into two varieties: the non-indurated, infecting chancre, or *chancroid*,—a term which is adopted in this work—and the indurated, true chancre. The one is never followed by constitutional taint, and frequently is followed by suppurating bubo; the other is the only *primary* sore which can give rise to secondary symptoms, and is rarely followed by other than indolent buboes. He believes that the virus of these ulcers are *distinct* poisons, which are incapable of confusion, or in other words, that the type of these ulcers remains unchanged in passing from one person to another—and after on able discussion of the views of MM. Bassereau, Clére, Diday, Ricord and others, upon the subject of the unity or duality of the poison, he gives the following corollaries:

"1. Among persons free from previous syphilitic taint, each of the two species of chancre is transmitted in its kind, the simple chancre as a simple chancre, limited in its action to the neighborhood of its site; the infecting chancre as an infecting chancre, followed by constitutional manifestations."

"2. A primary sore, with a soft base, and unaccompanied by induration of the neighboring lymphatic ganglia, in a subject already infected with syphilis, will, when communicated to a person free from syphilitic taint, give rise to a soft or hard chancre, according to the nature of the virus, which occasioned the first mentioned ulcer."

"3. The virus of the non-infecting chancre, is a poison distinct from that of the infecting chancre."

"4. Phagedenic ulceration of a chancre does not depend upon a specific difference in the virus."

Whilst allowing two kinds of *chancrous* virus, it does not follow that there are two kinds of syphilitic virus. The term syphilis, being limited in its meaning to a general disease, as well as local, only embraces the indurated chancre, whilst the soft or *chancroid*, is to be studied apart, as a non-infecting chancre; and as distinct a disease as gonorrhœa. Upon the subject of incubation, our author differs from Ricord; whilst, with him, he agrees that the induration of the chancre is the effect of a general infection of the system, he differs so far as to believe that the infecting chancre, from the earliest period of its existence, is not a local, but a constitutional lesion. If this is true, the importance of a correct diagnosis in all cases cannot be over-estimated, nor can any surgeon justify himself in remaining satisfied that he has saved his patient from constitutional disease, by treating the primary lesion with escharotics, to convert it into an innocuous ulcer, before the chancre is truly formed. On the other hand, the patient is to be saved from a course of mercury, when subject to a simple chancre only. The author does not believe in the abortive treatment of infecting chancre.

The chapters on the diagnosis and general treatment ot chancre and syphilis, are particularly instructive and suggestive; the author is firm in his faith of the value and necessity of a mercurial treatment, early commenced and well-continued in secondary syphilis.

The chapters on syphilization, tertiary syphilis, affections of the skin, mucous surfaces, etc., are as practical as can be desired, and embrace all that can be wanted by the student or practitioner in a text-book or work of reference.

The book is a credit to the author, and a valuable addition to American medical literature.

